# Oral lesions associated with dental implants: a retrospective study

**DOI:** 10.1186/s12903-025-06931-1

**Published:** 2025-09-29

**Authors:** Seyed Sepehr Mirebeigi-Jamasbi, Fazele Atarbashi-Moghadam, Saede Atarbashi-Moghadam

**Affiliations:** 1 Research Committee, School of Dentistry, Shahid Beheshti University of Medical Sciences, Tehran, Iran; 2 Department of Periodontology, School of Dentistry, Shahid Beheshti University of Medical Sciences, Tehran, Iran; 3 Department of Oral and Maxillofacial Pathology, School of Dentistry, Shahid Beheshti University of Medical Sciences, Tehran, Iran

**Keywords:** Dental implants, Oral cavity, Peri-implantitis, Reactive lesions, Neoplasms, Classification

## Abstract

**Background:**

Oral rehabilitation using dental implants has become one of the most effective options for treating patients needing tooth replacement. However, there are complications such as lesions associated with dental implants which can lead to severe consequences. This study aims to evaluate the frequency and microscopic type of lesions related to dental implants.

**Methods:**

This retrospective cross-sectional study included patients with documented lesions around dental implants referred to the Oral and Maxillofacial Pathology Department of Shahid Beheshti University of Medical Sciences between 2013 and 2024. Archived records were reviewed, and data on patient demographics, lesion location, and histopathological diagnosis were collected. Statistical analyses were conducted using SPSS20 employing chi-squared, fisher’s exact, One-way ANOVA, and independent samples T-test.

**Results:**

Out of 3641 biopsies, 43 (1.18%) cases had lesions associated with dental implants. Their mean age was 54.93 ± 14.47 years, with a male-to-female ratio of 1.38. Based on histopathologic characteristics of the lesions, they were classified into four categories (1) inflammatory/reactive, (2) developmental cysts, (3) neoplastic, and (4) oral potentially malignant disorders. The most frequent lesions belong to the inflammatory/reactive group and include non-specific inflammation (peri-implantitis) (39.5%) and peripheral giant cell granuloma (14%). Oral squamous cell carcinoma was the most frequent malignancy around dental implants.

**Conclusions:**

Various lesions have been observed around dental implants. Some of these lesions pose serious risks to the patient’s health, while others can be easily controlled if diagnosed promptly. It is highly recommended to analyze the peri-implant tissue histopathologically if the treatment option is surgical intervention.

**Graphical abstract:**

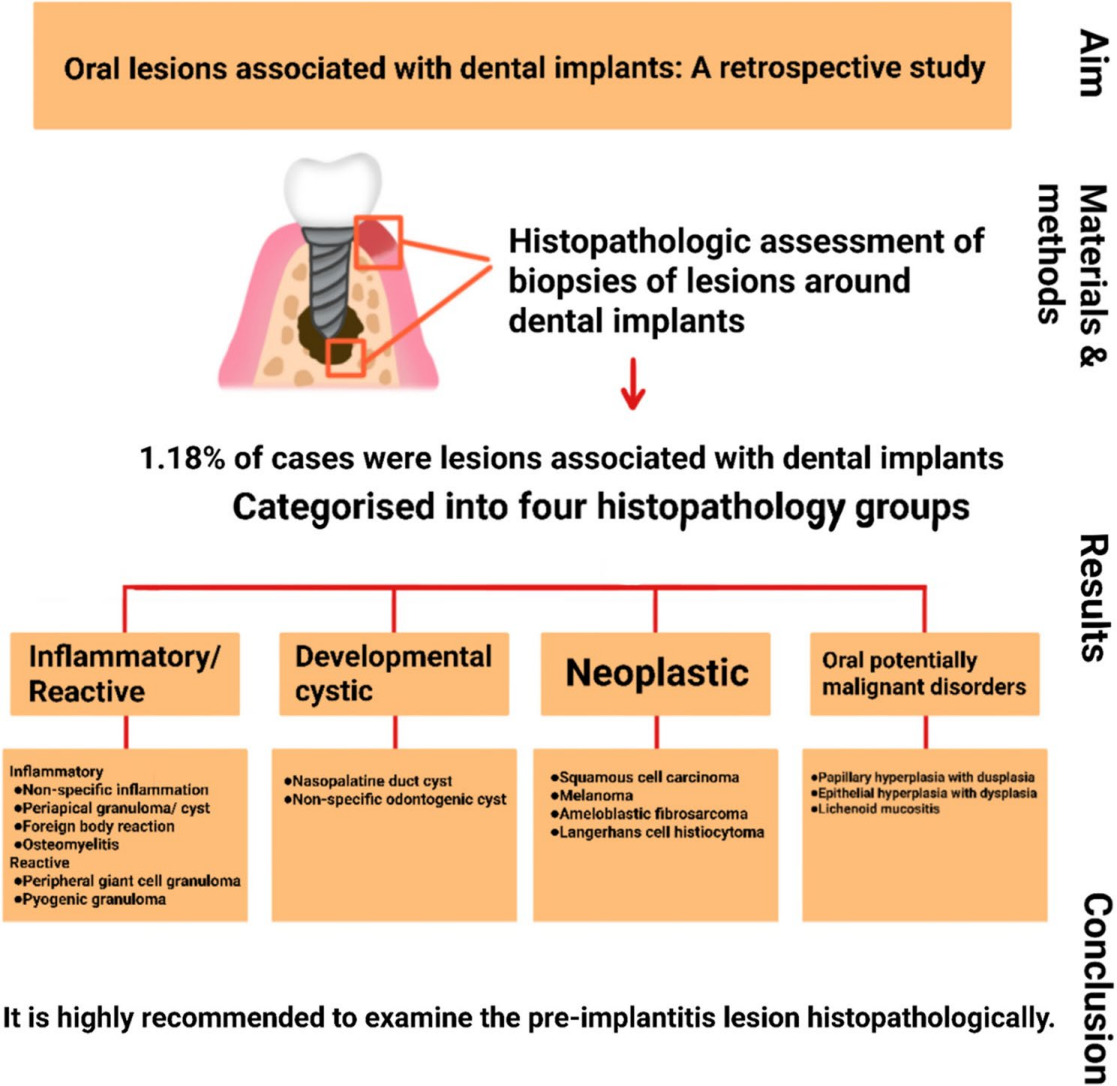

## Background

Oral rehabilitation utilizing osseointegrated dental implants has emerged as a premier choice for treating patients with tooth loss. This treatment option is regarded by some experts as the sole available effective treatment method. However, the widespread use of dental implants has also led to an increase in reported complications in the literature [1]. Common biological complications are peri-implant mucositis and peri-implantitis (PI) [2]. Periapical lesions around the apex of dental implants, known as apical peri-implantitis or retrograde peri-implantitis, have also been reported [3]. It can be stated that inflammatory processes represent the most prevalent complications associated with dental implants [1, 4]. The PI can clinically represent erythema or swelling of peri-implant mucosa, presence of bleeding on provocation, and/or suppuration [5, 6].

Reactive lesions around dental implants are another significant biological complication that requires careful attention as they can result in implant removal and failure [2]. Pyogenic granuloma (PG) and peripheral giant cell granuloma (PGCG) are the most frequent reactive lesions associated with dental implants [2]. These lesions, especially PGCG, can cause bone loss around dental implants and may lead to dental implant failure [7]. These Reactive lesions associated with dental implants may share clinical and radiographic features resembling PI but usually do not respond to standard treatment approaches, necessitating further histopathological evaluation [8]. Although the etiology of the development of these lesions around the dental implant has not been properly established, poor oral hygiene, poorly adapted prosthesis, chronic inflammation, and corrosion of dental implants were assumed as predisposing factors [2, 7].

Neoplastic lesions around dental implants also have been reported and sometimes mistakenly diagnosed as PI [6, 9]. Rarely, dental implants have been associated with primary or metastatic malignancies that closely mimic PI [6, 8]. Oral squamous cell carcinoma (OSCC) constitutes the majority of malignant lesions observed around or near dental implants [9]. The risk factors of neoplastic lesions around dental implants still have not been identified [10]. However, given their potential to mimic PI, particularly in the early stages, it is essential to conduct accurate diagnoses and maintain regular patient monitoring [9].

Although the use of dental implants has grown significantly, studies on the frequency and classification of implant-associated lesions remain limited. Understanding the prevalence and characteristics of these lesions is essential for helping practitioners distinguish peri-implantitis from other conditions, enabling more accurate diagnoses, treatment decisions, and the prevention of complications such as implant failure or delayed malignancy detection [8, 11]. Therefore, this study aims to address this gap by investigating the frequency of implant-associated lesions in the major oral pathology center in Iran over 11 years and present a histopathologic classification of these lesions.

## Methods

This retrospective study was ethically approved by the ethics committee of Shahid Beheshti University of Medical Sciences (IR.SBMU.DRC.REC.1402.018). Biopsies of patients with lesions associated with dental implants were assessed from records at the Oral and Maxillofacial Pathology Department, Shahid Beheshti University of Medical Sciences, Tehran, Iran center, over an eleven-year period (from April 2013 to April 2024). All patients with documented lesions involving dental implants, where the implant was surrounded by or in direct contact with the lesion, were included. Lesions that lacked direct contact with dental implant were excluded. Incomplete records or cases lacking a definitive histopathological diagnosis were also excluded. The study involved a complete census approach, reviewing all available cases within the specified period.

The study variables included the patient’s age (years) and gender (documented phenotype), location of lesions (jaws), and microscopic diagnosis. Microscopic sections (H&E stained) of implant-associated samples were re-evaluated and their diagnoses were confirmed by an oral pathologist. To avoid duplicate entries, recurrent cases were identified and reported as lesion recurrence. Additionally, the patients’ medical and dental histories were reviewed to evaluate whether the neoplastic lesions were primary or metastatic. The microscopic subgroups were also classified.

### Statistical analysis

Descriptive statistics were expressed as percentages and for qualitative variables, chi-squared and Fisher’s exact tests were employed. One-way ANOVA and independent samples T-test were used for age analysis. Analyses were performed using SPSS version 20. We considered *P* < 0.05 as statistically significant.

## Results

Out of 3,641 biopsies documented in the aforementioned center between 2013 and 2024, 43 patients (1.18%) presented with lesions associated with dental implants. Among these patients, 25 (58.1%) were male and 18 (41.9%) were female (male/female = 1.38). The age range was 19–84 years, with an average age of 54.9 ± 14.4 years. The most common age decades for lesions were the sixth, followed by the fifth and seventh decades. The characteristics of the included cases are presented in Table 1. Two cases were previously reported in the literature [9, 12].

**Table 1:** General characteristics of studied lesions

Data		*N*	%
Gender	Female	18	41.9
	Male	25	58.1
Involved Jaws*	Maxilla	18	42.9
	Mandible	24	57.1
Documented clinical symptom**	Exophytic mass	13	30.2
	Implant mobility/failure	8	18.6
	Bone expansion	4	9.3
	Pain	2	4.6
	White discoloration	2	4.6
	Ulcer	1	2.3

* In one case the involved jaw was not recorded.

** Some cases lacked clinical symptom records.

**Fig. 1:**
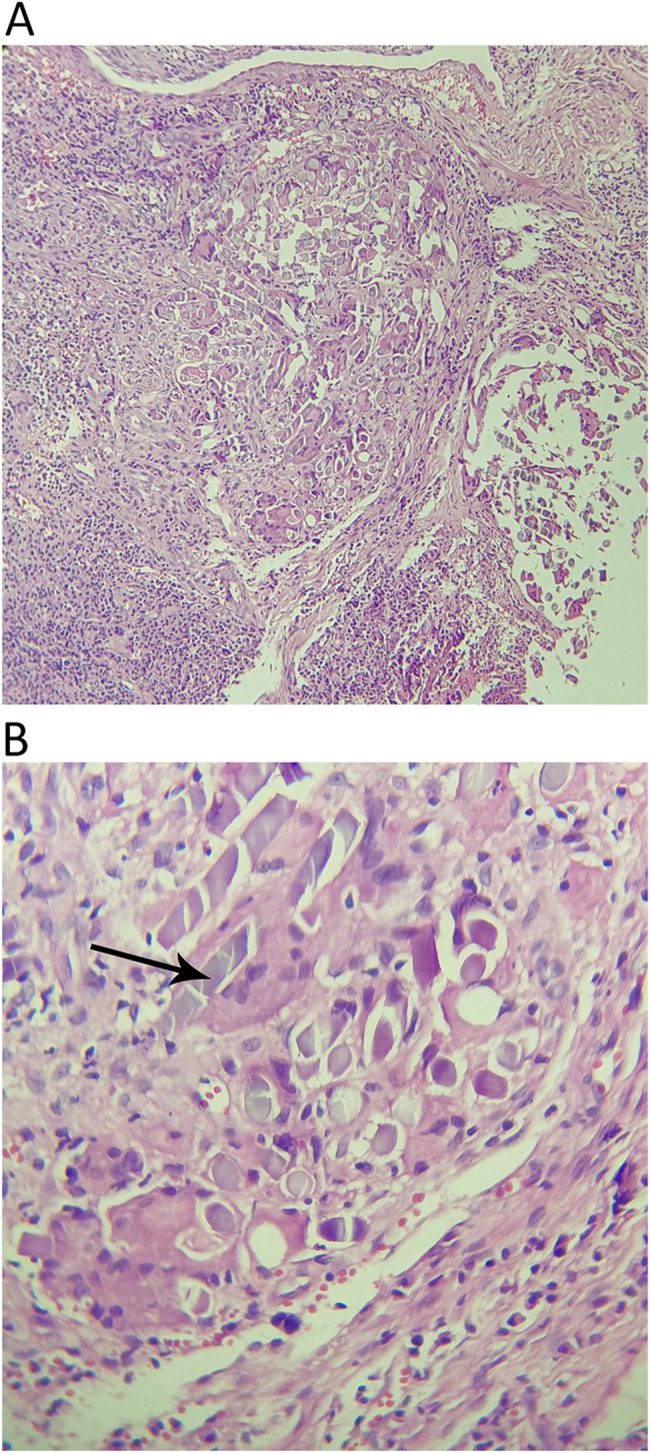
Histopathologic section (H&E staining) of a case of foreign body reaction. **A** Infiltration of chronic inflammatory cells (×100). **B** Higher magnification shows granulomatous inflammation which consists of multinucleated giant cells surrounding suture thread (black arrow) (×400)

**Fig. 2:**
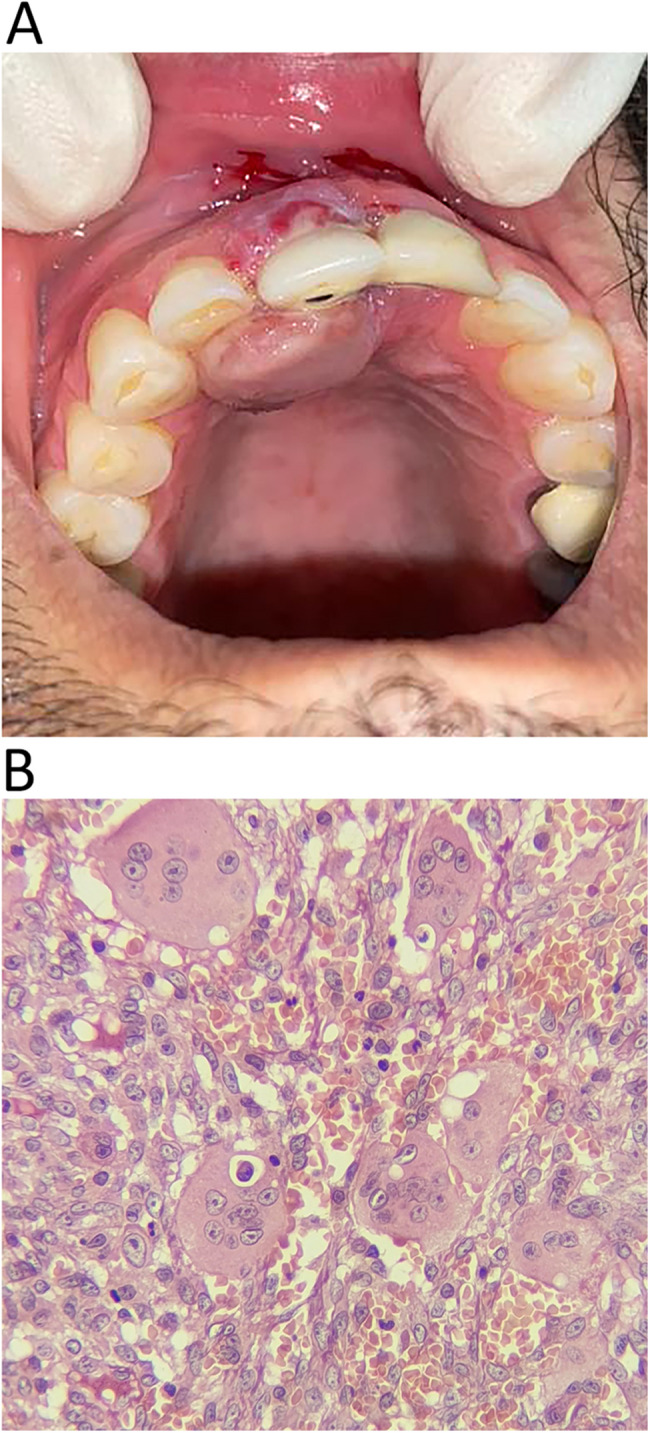
A case of peripheral giant cell granuloma (PGCG) associated with dental implants. **A** Palatal aspects of gingival mass around maxillary central implants. **B** Histopathologic section shows multinucleated giant cells in a spindle cell background. (H&E, ×400)

**Fig. 3:**
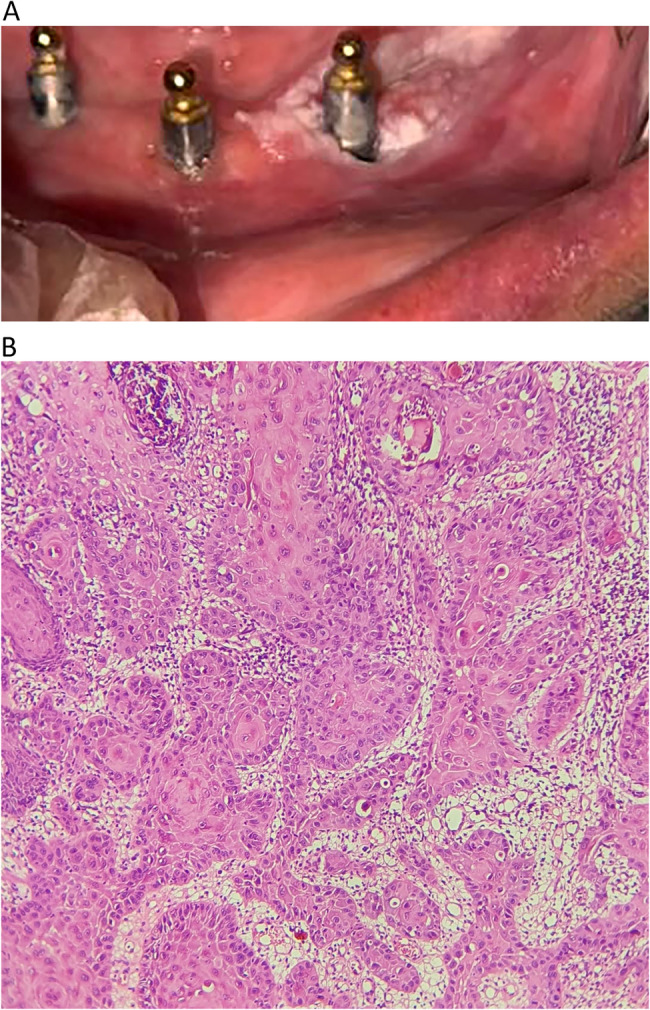
A case of oral squamous cell carcinoma (OSCC) associated with dental implant. **A** A white plaque at alveolar ridge and around dental implant in the anterior of mandible. **B** Histopathologic section shows islands of dysplastic squamous cells in fibrous stroma (H&E, ×400)

In the present study, 15 microscopic types of lesions were identified. The most common lesions were non-specific inflammation (17 cases, 39.5%), PGCG (6 cases, 14%), followed by OSCC, and apical granuloma (each with 3 cases, 7%). Based on Histopathologic characteristics of the lesions, they were classified into four categories (1) inflammatory/reactive, (2) developmental cysts, (3) neoplastic, and (4) oral potentially malignant disorders (OPMDs). There were 29 lesions in the inflammatory/reactive group (67.4%) and six were neoplastic (14%). Each OPMD and cystic group comprised 9.3% of the lesions (four for each group). The detailed information of each group is presented in Table 2. The inflammatory/reactive group comprises inflammatory and reactive lesions. From them non-specific inflammation (PI), apical granuloma (retrograde PI), foreign body reaction (Fig. 1), and osteomyelitis were inflammatory and PGCG (Fig. 2) and PG were reactive. Developmental cysts group include nasopalatine duct cyst (NPDC) and non-specific odontogenic cysts. A case of NPDC had a recurrence 3 years after treatment. The neoplastic group contains OSCC (Fig. 3), melanoma, ameloblastic fibrosarcoma, and Langerhans cell histiocytosis (LCH). All of these neoplastic lesions were primary tumors. A case of ameloblastic fibrosarcoma had a history of ameloblastic fibroma in the area which was treated 5 years before. The OPMDs group comprises papillary hyperplasia with dysplasia, epithelial hyperplasia with dysplasia, and ulcerative lichenoid mucositis. Among all lesions, 38 (88.4%) were benign, and 5 (11.6%) were malignant.

**Table 2:** Demographic data of cases based on histopathologic subgroup

Lesion group		Histopathologic diagnosis	N (%)	Gender		Mean age (years)	Involved jaws*	
				Female (N)	Male (N)		Maxilla (N)	Mandible (N)
Inflammatory/reacti-ve group	Non-specific inflammation (PI)		17 (39.5)	7	10	52.2	7	9
	Periapical granoluma (Retrograde PI)		3 (7)	2	1	61.7	3	-
	Osteomyelitis		1 (2.3)	1	-	31	-	1
	Foreign body reaction		1 (2.3)	1	-	54	-	1
	PGCG		6 (14)	2	4	50.5	2	4
	PG		1 (2.3)	-	1	42	-	1
Developmental cysts group	NPDC		2 (4.7)	-	2	63	2	-
	Non-specific odontogenic cyst		2 (4.7)	1	1	41.5	2	-
Neoplastic group	OSCC		3 (7)	2	1	71.7	-	3
	Malignant melanoma		1 (2.3)	-	1	75	1	-
	Ameloblastic fibrosarcoma		1 (2.3)	-	1	32	-	1
	LCH		1 (2.3)	1	-	61	-	1
oral potentially malignant disorders group	Papillary hyperplasia with dysplasia		2 (4.7)	-	2	69	1	1
	Epithelial hyperplasia with dysplasia		1 (2.3)	-	1	70	-	1
	Ulcerative lichenoid mucositis		1 (2.3)	-	1	60	-	1

*LCH* Langerhans cell histiocytosis, *NPDC* Nasopalatine duct cyst, *OSCC* Oral squamous cell carcinoma, *PG* Pyogenic granuloma, *PGCG* peripheral giant cell granuloma, *PI* Peri-implantitis.

* Involved jaw was not recorded in one case in the PI group.

One-way ANOVA and independent samples T-test were conducted for age analyses and revealed a statistically significant difference, indicating that patients with OPMDs and neoplastic lesions had a higher mean age (67 and 63.8 years, respectively) compared to those with inflammatory/reactive and cystic lesions, whose mean age was 51.8 and 52.2 years, respectively (*P* = 0.04). Patients with malignant lesions had a mean age 10.7 years higher than those with benign lesions (*p* = 0.16). Chi-squared and Fisher’s exact tests revealed no significant differences between the groups when comparing them by sex and location (*P* > 0.05).

## Discussion

In the present study, conducted over 11 years, 43 dental implant-associated lesions were observed, representing 1.18% of all patients. Paparella et al. reported 68 samples through 28 years, amounting to 0.17% of all cases [13]. Sotorra-Figuerola et al. gave an account of 5.9% (111 cases) during 18 years [11]. Therefore, dental implant-associated lesions include a small number of oral lesions. However, with the growing use of this treatment modality for replacing missing teeth, implant-associated lesions are expected to be increased [14]. As in the study of Paparella et al. [13], which included the data from the 90 s, the implant-associated lesions were much less. The mean age of patients in this study was 54.9 years, with a tendency towards the sixth decade of life, compared to 55.2 [8], 59 [11], and 60 [13] years in other studies. Dental implants are used to replace missing teeth; therefore, the higher frequency of dental implant-associated lesions in higher ages (compared with the lesions in cases without dental implants) seems normal. Furthermore, in the current study, the frequency of malignant lesions and OPMDs associated with dental implants was found to be significantly higher than that of lesions not associated with implants. A higher frequency of cases was observed in men, contrary to other studies [8, 11, 13]. The mandible accounted for most of the lesions in this study, which is in agreement with other studies [11, 13]. However, Kaplan et al. reported a slight maxillary predilection [8].

Fifteen microscopic types of lesions were identified in this study and classified into four categories: (1) inflammatory/reactive, (2) developmental cysts, (3) neoplastic, and (4) OPMDs. The other lesions that have been reported in the other studies [8, 11, 13] can also be included in this classification.

## Inflammatory/reactive group

### Inflammatory lesions

Similar to previous studies [8, 11, 13], inflammatory/reactive lesions are the most common lesions associated with dental implants in this research. The main inflammatory lesion was non-specific inflammation (39.5%) clinically known as PI, in agreement with other studies [8, 11]. In the current study, histopathologic sections showed non-specific inflammation mostly composed of lymphocyte and plasma cell infiltration intermixed with neutrophils. Plaque biofilm is considered the key etiological factor of this condition and patients with poor plaque control are at high risk [15]. Other possible contributing factors include the amount of keratinized gingiva, occlusion status, prosthetic design, surgical procedures, history of periodontal disease, and smoking [5, 16]. Regular supportive periodontal treatment sessions are a primary preventive measure against these inflammatory lesions [16]. Kaplan et al., have reported the actinomyces-related inflammation which exhibited bacterial colonies of actinomyces within the inflamed peri-implant tissues [8].

Apical granuloma associated with dental implants, clinically called retrograde PI, is another inflammatory lesion that comprised 7% of the cases. These lesions are primarily associated with endodontic infection from a previously treated or adjacent tooth [3, 17, 18]. Other contributing factors include contamination of the surgical area and implant surface, elevated bone temperature during drilling, immediate implant placement post-extraction, residual roots, and proximity of the implant to neighboring teeth [3]. If antibiotic treatment alone did not cure the infection, surgical intervention would be necessary [19, 20]. It is recommended to assess the tissue histopathologically, to rule out the presence of other lesions [8]. Chronic abscess formation or implant instability may require implant removal. Early detection and intervention are critical in managing these lesions effectively [19].

Foreign body reaction is an inflammatory tissue response to implantation of foreign material [21]. Microscopically, the most common type of foreign body reaction would be granulomatous inflammation and formation of multi-nucleated foreign body giant cells [22], although encapsulation of the implanted material in a layer of fibrous tissue may also be seen [21]. Many multinucleated giant cells and suture material were found around the dental implant in the foreign body reaction case in this study.

Osteomyelitis is a rapidly progressing infection of bone that affects the local blood supply and results in necrosis of the bone and sequesters formation [14]. Kellesarian et al. in a systematic review showed the increasing prevalence of osteomyelitis associated with dental implants after the year 2013 and they showed a predilection to women and mandible [14]. Generally, the prevalence of osteomyelitis is higher among middle-aged and in the mandible [23]. Therefore, the prevalence of osteomyelitis associated with dental implants followed the pattern of osteomyelitis of the jaws. In this study, there was also a case of osteomyelitis in the mandible of a woman. Kellesarian et al. [14] reported that more than half of the patients presented systemic or environmental conditions including diabetes mellitus, bisphosphonate treatment, and smoking. To prevent infection from spreading, radical and rapid treatment should be considered. Moreover, in most osteomyelitis associated with dental implant cases implant removal was inevitable [14].

### Reactive lesions

PGCG and PG are the most common reactive lesions associated with dental implants. Both lesions were observed in this study, with PGCG being more frequent than PG. Most of these cases were found in males (71.4%). This finding follows Roman-Quesada et al. [7] that concluded reactive lesions associated with dental implants were more prevalent in men in contrast to peri-dental reactive lesions. They related this finding to lower oral hygiene and plaque control in men. These lesions are typically exophytic masses clinically, but lesions resembling PI were also reported [8]. Reactive lesions are soft tissue lesions that rarely affect the underlying bone. Nevertheless, reactive lesions associated with dental implants can cause bone loss and implant failure [2, 7]. In this study, three cases of reactive lesions resulted in implant mobility and/or failure while resembling peri-implantitis or ulcers clinically.

## Developmental cysts group

NPDC is thought to arise from the proliferation of epithelial remnants within the nasopalatine duct, triggered by local trauma, infection, or spontaneously [24, 25]. Radiographically, the differential diagnosis includes radicular cysts and odontogenic keratocysts. However, radicular cysts are associated with non-vital teeth. Additionally, calcified odontogenic cysts and adenomatoid odontogenic tumors may be considered in the differential diagnosis if they do not exhibit radiopaque areas [26]. NPDC associated with dental implant occurs when a dental implant is placed in the vicinity of the nasopalatine canal or the canal is traumatized during implant drilling [25]. Designing a precise treatment plan with an appropriate distance from the nasopalatine canal, and using cone beam computed tomography to indicate the exact location of the canal help to avoid this lesion around the dental implant [25]. Typically, removing the implant along with the cyst is recommended. However, in cases where the implant remains stable and the lesion does not affect neighboring teeth or implants, some suggest retaining the implant [24, 25]. In the current study, preserving the implant led to lesion recurrence in one patient three years after the initial diagnosis.

In this study, two non-specific odontogenic cysts were found that did not show characteristic microscopic findings. Both of them showed nonkeratinized stratified squamous epithelium. It is hypothesized that these cysts may have already been present in the jawbone before implantation or the dental implants were placed near the follicle of un-erupted teeth. Paparella et al. [13], also found inflammatory and developmental cysts along with a case of simple bone cyst.

## Neoplastic group

SCC is the most common malignancy of the oral cavity [1]; therefore, it is not surprising that most of the malignancies around dental implants were OSCC. The role of dental implants in OSCC formation remains uncertain, with hypotheses including damage to periodontal tissues and chronic inflammation promoting cell proliferation and genetic instability [1, 4, 6, 27]. Other theories involve corrosion products, metal ion release, and migration of malignant cells through the peri-implant gingival sulcus [28]. Clinical symptoms of SCC around dental implants were various; such as painful ulcers, swelling, bone loss, and lesions resembling PI [4, 6, 8]. Prevention strategies include assessing individual risk factors before implant placement, biopsy of suspicious oral lesions, and regular post-treatment monitoring [1, 27]. Metastases around dental implants were also reported [29]. In patients with a history of malignancy, careful consideration of implant treatment is advised and the possible risks should be informed to the patients [9]. Furthermore, the case of ameloblastic fibrosarcoma analyzed in this study had a history of ameloblastic fibroma in the same area about five years ago which recurred in a malignant form.

The association of LCH with dental implants has not been mentioned in the literature earlier. But other hematopoietic disorders such as large B-cell lymphoma or plasmacytoma have been reported in the vicinity of dental implants [6].

### OPMDs group

In this study, a case of lichenoid mucositis was observed adjacent to a mandibular implant in a 60-year-old man. Rasul et al. reported a similar case of lichen planus near a gold implant in a 64-year-old woman, attributing it to prolonged mucosal irritation [30]. Paparella et al. reported two cases of lichenoid lesions in their cases [13]. The precise cause of lichen planus around dental implants remains uncertain. However potential factors include inducing an immune response with the participation of activated macrophages that secrete cytokines, facilitating the inflammatory process [31]. Another theory is dysfunction of T cells, as seen in contact dermatitis, which can lead to lichen planus, characterized by delayed hypersensitivity reactions [30]. In current study two cases of epithelial dysplasia were seen. Noguchi et al. [27] reported a case of carcinoma in situ around dental implant in a 65-year-old woman without the major risk factors for oral SCC. They suggested that the persistence of peri-implant mucositis or PI around the dental implant was considered as an acceptable risk factor for carcinogenesis.

Finally, this is a retrospective histopathologic study with its limitations. Unfortunately, we could not access some clinical and radiographic data of patients. Since surgeons have not always sent the tissue which was removed during the surgical treatment of peri-implantitis for histopathologic analysis, there are limited documents of lesions associated with dental implants.

## Conclusions

Various lesions have been observed around dental implants. Some of these lesions pose serious risks to the patient’s health, while others can be easily controlled if diagnosed promptly. However, delayed diagnosis can lead to dental implant failure and necessitate explantation. One of the important aims of maintenance care in dental implant treatment must be early diagnosis of these conditions. It is highly recommended to analyze the peri-implant tissue histopathologically if the treatment option was surgical intervention, especially in recurrent cases or cases that do not respond to non-surgical treatments.

## Data Availability

No datasets were generated or analysed during the current study.

## Abbreviations

PG: Pyogenic granuloma
PGCG: Peripheral giant cell granuloma
PI: Peri-implantitis
OSCC: Oral squamous cell carcinoma
OPMD: Oral potentially malignant disorders
NPDC: Nasopalatine duct cyst
LCH: Langerhans cell histiocytosis
